# The Challenge of Managing Atrial Fibrillation during Pregnancy

**DOI:** 10.31083/j.rcm2410279

**Published:** 2023-10-07

**Authors:** Fabiana Lucà, Fabrizio Oliva, Maurizio Giuseppe Abrignani, Maria Giovanna Russo, Iris Parrini, Stefano Cornara, Roberto Ceravolo, Carmelo Massimiliano Rao, Silvia Favilli, Andrea Pozzi, Simona Giubilato, Stefania Angela Di Fusco, Berardo Sarubbi, Raimondo Calvanese, Alaide Chieffo, Sandro Gelsomino, Carmine Riccio, Massimo Grimaldi, Furio Colivicchi, Michele Massimo Gulizia, on behalf of the Management and Quality Working Group, Pediatric Cardiology Working Group, and Arrhythmias Working Groups ANMCO

**Affiliations:** ^1^Cardiology Department, Grande Ospedale Metropolitano, GOM, AO Bianchi Melacrino Morelli, 89124 Reggio Calabria, Italy; ^2^Cardiology Unit, ASST Grande Ospedale Metropolitano Niguarda, 20162 Milano, Italy; ^3^Cardiology Department, P.O. Paolo Borsellino Hospital, ASP Trapani, 91025 Marsala (TP), Italy; ^4^Pediatric Cardiology Unit, Monaldi Hospital, University of Campania L. Vanvitelli, 80138 Naples, Italy; ^5^Cardiology Department, Mauriziano Hospital, 10128 Torino, Italy; ^6^Cardiology Department, Ospedale San Paolo, 17100 Savona, Italy; ^7^Cardiology Unit, Giovanni Paolo II Hospital, 88046 Lamezia, Italy; ^8^Department of Pediatric Cardiology, Meyer Hospital, 50139 Florence, Italy; ^9^Department of Cardiology, Ospedale Valduce, 22100 Como, Italy; ^10^Cardiology Department, Cannizzaro Hospital, 95123 Catania, Italy; ^11^Clinical and Rehabilitation Cardiology Department, San Filippo Neri Hospital, ASL Roma 1, 00135 Roma, Italy; ^12^Adult Congenital Heart Diseases Unit, AORN dei Colli, Monaldi Hospital, 80131 Naples, Italy; ^13^Cardiology Department, Ospedale del Mare, 80147 Napoli, Italy; ^14^Interventional Cardiology Unit, IRCCS San Raffaele Scientific Institute, 20132 Milan, Italy; ^15^Cardiothoracic Department, Maastricht University Hospital, 6229 Maastricht, The Netherlands; ^16^Cardiovascular Department, Sant'Anna e San Sebastiano Hospital, 81100 Caserta, Italy; ^17^Department of Cardiology, General Regional Hospital “F. Miulli'', Acquavivadelle Fonti, 70021 Bari, Italy; ^18^Cardiology Department, Garibaldi Nesima Hospital, 95122 Catania, Italy

**Keywords:** atrial fibrillation (AF), pregnancy, electrical cardioversion (ECV), antiarrhythmic drugs (AADs), anticoagulants, Pregnancy Heart Team

## Abstract

The incidence of atrial fibrillation (AF) during pregnancy increases with maternal age and with the presence of structural heart disorders. Early diagnosis and prompt therapy can considerably reduce the risk of thromboembolism. The therapeutic approach to AF during pregnancy is particularly challenging, and the maternal and fetal risks associated with the use of antiarrhythmic and anticoagulant drugs must be carefully evaluated. Moreover, the currently used thromboembolic risk scores have yet to be validated for the prediction of stroke during pregnancy. At present, electrical cardioversion is considered to be the safest and most effective strategy in women with hemodynamic instability. Beta-selective blockers are also recommended as the first choice for rate control. Antiarrhythmic drugs such as flecainide, propafenone and sotalol should be considered for rhythm control if atrioventricular nodal-blocking drugs fail. AF catheter ablation is currently not recommended during pregnancy. Overall, the therapeutic strategy for AF in pregnancy must be carefully assessed and should take into consideration the advantages and drawbacks of each aspect. A multidisciplinary approach with a “Pregnancy-Heart Team” appears to improve the management and outcome of these patients. However, further studies are needed to identify the most appropriate therapeutic strategies for AF in pregnancy.

## 1. Introduction

Atrial fibrillation (AF) is widely recognized as the most common sustained tachyarrhythmia in adults [1], affecting approximately 44 million people worldwide. AF is also one of the most frequently reported cardiac arrhythmias during pregnancy, with an incidence of 27/100,000. Of note, this incidence has been increasing over the past decades [2, 3, 4].

Physiological changes in hormonal status and hemodynamics occur during pregnancy. These include plasma volume expansion, an increased heart rate (HR) at rest and during cardiac output, enhanced atrial stretching, and a dominance of parasympathetic over sympathetic activity [5]. These factors can predispose to cardiac arrhythmias [6] in women with or without structural heart disease [7, 8, 9, 10]. Importantly, the occurrence of AF during pregnancy is associated with an increased risk of maternal and fetal complications [11], including heart failure (HF) due to the hemodynamic imbalance [3, 12]. Moreover, a higher risk of thrombotic complications also arises in pregnancy due to increased procoagulant factors and reduced anticoagulation activity, thereby creating a state of hypercoagulability [13, 14, 15]. Hence, the management of AF during pregnancy remains a major challenge and requires accurate workup and a multidisciplinary approach.

The best therapeutic approach for AF during pregnancy remains to be established due to the scarce evidence and limited data available to date. This review presents a comprehensive discussion of the management of AF during pregnancy.

## 2. Incidence of AF and Clinical Risk Factors during Pregnancy

Supraventricular tachycardias (SVT), especially AF and atrial flutter (AFL), are the most common sustained arrhythmias during pregnancy [2, 8, 10, 16, 17, 18]. Certain factors such as advanced age (>41 years), African-American ancestry, and a lower socioeconomic status have been associated with the development of AF in pregnant females [2, 3, 19]. The wide range in prevalence of AF among different racial/ethnic groups may reflect a genetic predisposition [19]. Similar to other arrhythmias, AF is more frequent in black women than in white women [3]. Age is also a strong risk factor for AF [20], with the prevalence of AF increasing significantly after 40 years of age [19]. Compared to women aged <25 years, the odds ratio (OR) for AF in women aged 30–34, 35–39, and ≥40 years was reported as 4.1, 4.9, and 5.2, respectively [19].

**Fig. 1.**
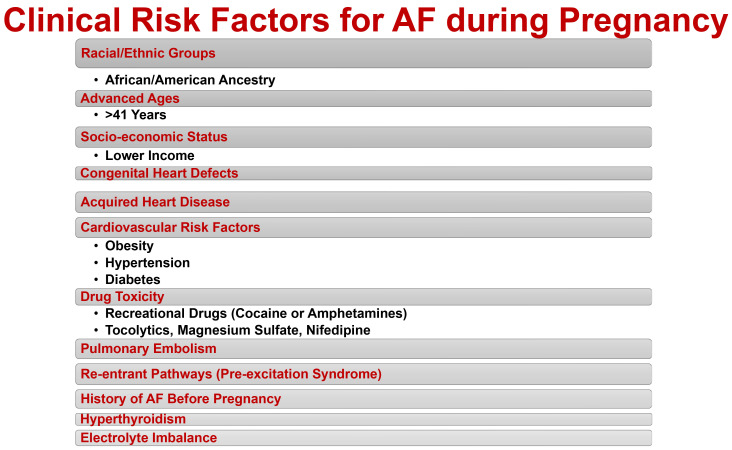
**Clinical risk factors for atrial fibrillation (AF) during pregnancy.** AF during pregnancy generally indicates an underlying congenital or acquired heart disease. Cardiovascular risk factors such as obesity, chronic hypertension and diabetes have been associated with AF during pregnancy. Moreover, AF is more frequent in older women (>41 years), women of African-American ancestry, and women with lower socioeconomic status. Drug toxicity, pulmonary embolism, accessory pathways, re-entrant circuits, hyperthyroidism, and electrolyte imbalance have also been associated with the development of AF during pregnancy.

The presence of congenital or acquired cardiovascular disease (CVD), and of cardiovascular (CV) risk factors [12, 21] has also been reported to increase the risk of AF [22]. Several studies have examined the relationship between AF during pregnancy and multiple clinical risk factors [7, 19, 21, 23, 24] (Fig. 1). The Registry of Pregnancy and Cardiac disease (ROPAC) study identified prior history of AF, beta-blocker consumption, aortic valve (AV) and mitral valve (MV) disease, and cardiomyopathies as risk factors for arrhythmic recurrence in pregnancy [16]. However, the occurrence of AF alone during pregnancy is extremely rare [7, 10, 19, 25, 26, 27, 28, 29, 30, 31, 32].

Only 2 cases of AF were reported among 2552 referrals to hospital for severe maternal CV complications in the Netherlands between 2004 and 2006 [33]. Analysis of the Groupe d’Étudeen Médecine Obstétricale du Québec (GÉMOQ) registry of women with a structurally normal heart [25] revealed 16 cases of AF (94% with paroxysmal AF), of which 81% showed spontaneous cardioversion usually within 24 hours. In a study of Kaiser Permanente Southern California hospital patients between 2003 and 2013, 157 AF cases were identified among 264,730 pregnancies (59.3/100,000) [19]. In a systematic review of 7 cohort studies comprising 301,638 cases, the pooled estimated incidence of AF in pregnancy in women with or without structural heart disease CVD was 2.2% and 0.3%, respectively [34]. AF is thus more frequent in women with underlying cardiac anomalies such as cardiomyopathy or congenital heart defects (CHDs) [7, 10], as also reported in case series and individual case reports [19, 26, 35, 36, 37, 38, 39, 40, 41]. The incidence of AF was also shown to correlate with the type and severity of valvular heart disease (VHD): 29% in isolated mitral stenosis (MS), 16% in isolated mitral regurgitation (MR), 52% in combined MS and MR, and 1% in aortic valvular disease [42].

In developing countries, AF is frequently observed in young females with widespread rheumatic heart disease [43, 44]. Szekely and Snaith [45] found pre-excited AF in 8% of pregnant women with rheumatic heart disease, compared to new onset AF in 2.5% of pregnant women. Khairy *et al*. [46] found no AF in a study of 90 pregnancies in 53 women with CHDs. Lee *et al*. [19] reported 226 cases of cardiac arrhythmias in 136,422 pregnant women hospitalized at a single center. Of these, three patients had episodes of AF (1% of all admissions, with a prevalence of 2/100,000 pregnancies), and all three patients had structural CHDs. In a retrospective analysis of 93 patients admitted with cardiac disease in Durban, South Africa, 9 women (9.7%) had AF, of which four had metallic valve prosthesis, four had severe MS, and one had mixed MV disease [47]. Of 1321 consecutive pregnant women with CHDs, VHD, coronary artery disease (CAD) or cardiomyopathy enrolled at 60 hospitals in the multinational Registry of Pregnancy and Cardiac disease (ROPAC), 17 (1.3%) developed AF during pregnancy [16]. Furthermore, women with MV disease showed a higher incidence (2.5%) than those with other cardiac lesions. AF occurred in 10 patients with MV disease (3%), four of whom had a history of valve surgery.

An incidence of 0.7% has been reported for AF in pregnant women with CHDs such as ventricular septal defects, atrioventricular septal defects, and Fontan circulation [16].

In the ROPAC study, only one patient with cardiomyopathy developed AF in the second trimester [16]. Previously reported cases of AF in pregnancy occurred in the third trimester, and especially during labor and delivery. These were mainly due to drugs such as terbutaline and nifedipine used for tocolysis, or as a manifestation of peripartum cardiomyopathy [20, 48].

The Kaiser Permanente study also found the risk of AF was higher during the third trimester than the first trimester (OR 3.2; 95% confidence interval [CI]: 1.5–7.7) [19]. In contrast, recent studies have reported a peak in AF during the second trimester [16, 37]. It is worth noting that the risk of recurrent AF in patients with previous arrhythmias has been estimated at 39.2%–52% [34, 37]. Hence, a history of AF before pregnancy is likely to be an independent predictor of AF during pregnancy [2, 16].

It has also been established that COVID-19 infection may predispose to arrhythmias, including AF, especially if there are coexisting CV risk factors and cardiac disorders [49, 50, 51].

## 3. Pathophysiological Mechanisms

**Fig. 2.**
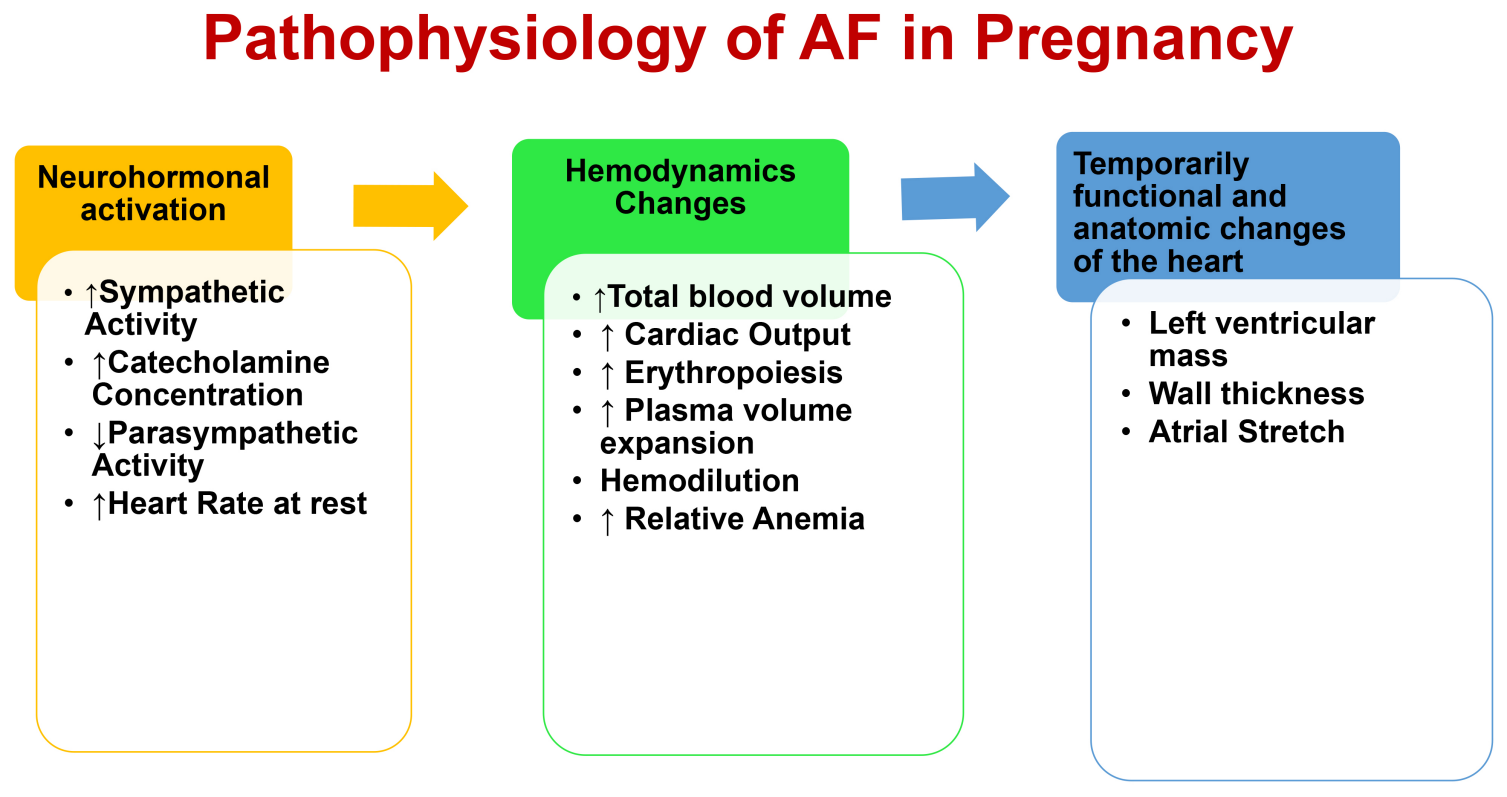
**Pathophysiology of atrial fibrillation (AF) in pregnancy.** Several neurohormonal and hemodynamic changes characterize pregnancy, including vasodilation, neurohormonal activation, enhanced sympathetic tone, and increased resting heart rate and total blood volume.

Several neurohormonal and hemodynamic adaptations occur in the maternal body during pregnancy [52] (Fig. 2). The major changes are vasodilation of the systemic arterial vasculature, neurohormonal activation, and increased total blood volume [53]. A stronger sympathetic response with enhanced sympathetic feedback to physiological stress has been observed during pregnancy, particularly in the third trimester [54, 55]. Therefore, the presence of a higher heart rate in pregnant women may be a predisposing factor for AF. Notably, an increased heart rate at rest is considered to be an arrhythmogenic marker of AF. Moreover, premature atrial and ventricular complexes are more frequent during pregnancy [55].

A decrease in peripheral vascular resistance occurs early in pregnancy and reaches the lowest value (about 40% below baseline) during the fourth and fifth months [56]. Nervous system sympathetic activity and heart rate show a parallel increase during normal pregnancy [5]. As a consequence, cardiac output increases by up to 50%. Along with the vascular and neurohormonal adaptations of the maternal body, changes in plasma volume and red cell mass also occur during gestation. Erythropoiesis and total blood volume increase, while concomitant plasma volume expansion causes “relative anemia” due to hemodilution [57].

Physiological changes in the vascular bed, neurohormonal balance, and fluid status affect both heart function and structure [53]. The left ventricular mass and wall thickness temporarily increase compared to pre-pregnancy values, together with mild four-chamber dilation, as observed by CV imaging studies in gestating women.

These temporary physiological changes during pregnancy may be predisposing factors for maternal cardiac dysrhythmias. Moreover, the combination of hemodynamic, hormonal, and autonomic alterations are thought to be arrhythmogenic determinants of AF in pregnant females [58, 59]. Notably, the intravascular volume expansion during pregnancy causes ventricular end-diastolic and volume atrial dilation, resulting in mechanical and electrical effects such as the stretching of atrial muscle cells, shortening of the atrial effective refractory period (AERP), and the slowing of electrical conduction [59, 60, 61]. Growth of adrenergic myocardial receptor density and responsiveness have been associated with increased levels of plasma estrogen and progesterone [59, 62]. Additionally, the gradual increase in 7 β-estradiol (E2) concentration during pregnancy contributes to the rising HR [63, 64, 65].

Increased catecholamine plasma levels, enhanced catecholamine sensitivity, and the prevalence of sympathetic activity have all been postulated as underlying mechanisms for AF during pregnancy [59]. Relaxin may also have a role in triggering AF during pregnancy due to its chronotropic action [66].

## 4. Outcomes of AF

The incidence and maternal/fetal outcomes of AF in pregnancy remain unclear. AF is known to be associated with good pregnancy outcomes in women with normal hearts [25]. In the Kaiser Permanente study, adverse maternal cardiac events were rare in AF patients, with just two women developing HF and no maternal deaths reported [19]. In the ROPAC study, women with AF had significantly higher maternal mortality than those without (11.8% vs. 0.9%; *p* = 0.01) [16]. Adverse fetal events occurred in 35% of patients with paroxysmal AF and in 50% of those with permanent AF [16]. In a systematic review, the pooled incidence of pre-eclampsia and congestive HF among pregnant women with AF was estimated to be 4.1% and 9.6%, respectively [34].

It is widely accepted that both AF and pregnancy can predispose women to thromboembolic complications. However, despite a majority of patients in the Kaiser Permanente study having a ${\mathrm{CHA}}_{2}$${\mathrm{DS}}_{2}$-VASc score of 1.2 ± 0.5, no strokes or systemic embolic events were observed. Nevertheless, it should be noted that one point in this score was due to female gender [19]. In the ROPAC study, one case with AF and MV disease died postpartum due to a presumed thromboembolic event. No other thromboembolic complications were reported [16]. In a cross-sectional study that included 81,983,216 pregnancy hospitalizations from 1994–2011 in a U.S. Nationwide Inpatient Sample, AF substantially increased the stroke risk in cases of pregnancy hospitalization for hypertensive disorders [67].

## 5. Effects of AF on Fetal Conditions during Pregnancy

AF during pregnancy affects not only the maternal outcome, but also has important consequences for the fetus. It is well established that AF is associated with higher rates of maternal mortality (MM) and lower fetal birth weight [22, 68, 69].

Depending on the gestational period, the potential teratogenic effect of drugs can negatively influence fetal development, organogenesis and growth. Moreover, the fetal outcome is deemed to be poor if a hemodynamic impairment occurs [22, 68, 69].

In the ROPAC study [16], AF and AFL were observed in 17 of 1321 (1.3%) pregnant females with structural CVD, whilst the remaining 1304 patients were in sinus rhythm (SR). A higher MM has been reported in women with AF/AFL compared to recipients in SR. The mean gestation period was shorter in women with AF/AFL than those in SR (37.5 vs. 38.0 weeks, *p *= 0.25). Delivery by cesarean section was more frequent in women with AF/AFL than in those without (47% vs. 41%, *p *= 0.58). No fetal or neonatal deaths occurred in AF/AFL patients [16]. Low birth weight (<2500 g) was significantly more frequent in women with AF/AFL than in those without (35% vs. 14%; *p* = 0.02). Fetal complications included premature birth [16].

Intrauterine growth retardation occurred in 17.6% and 5.6% of patients in the AF/AFL and SR groups, respectively. Premature birth (<37 weeks) occurred more often in patients with AF/AFL (29% vs. 15%; *p *= 0.16). The adjusted mean birth weight was significatively lower in women with AF than those without (3026 g vs. 3358 g; *p <* 0.001). In a study of 264,730 pregnant women that included 157 with AF, the admission rate to the neonatal intensive care unit was higher in patients with AF (17/157, 10.8%) than in those without (13,309/264,573, 5.1%; *p *= 0.003) [22].

## 6. Rhythm Control and Electrical Cardioversion

**Fig. 3.**
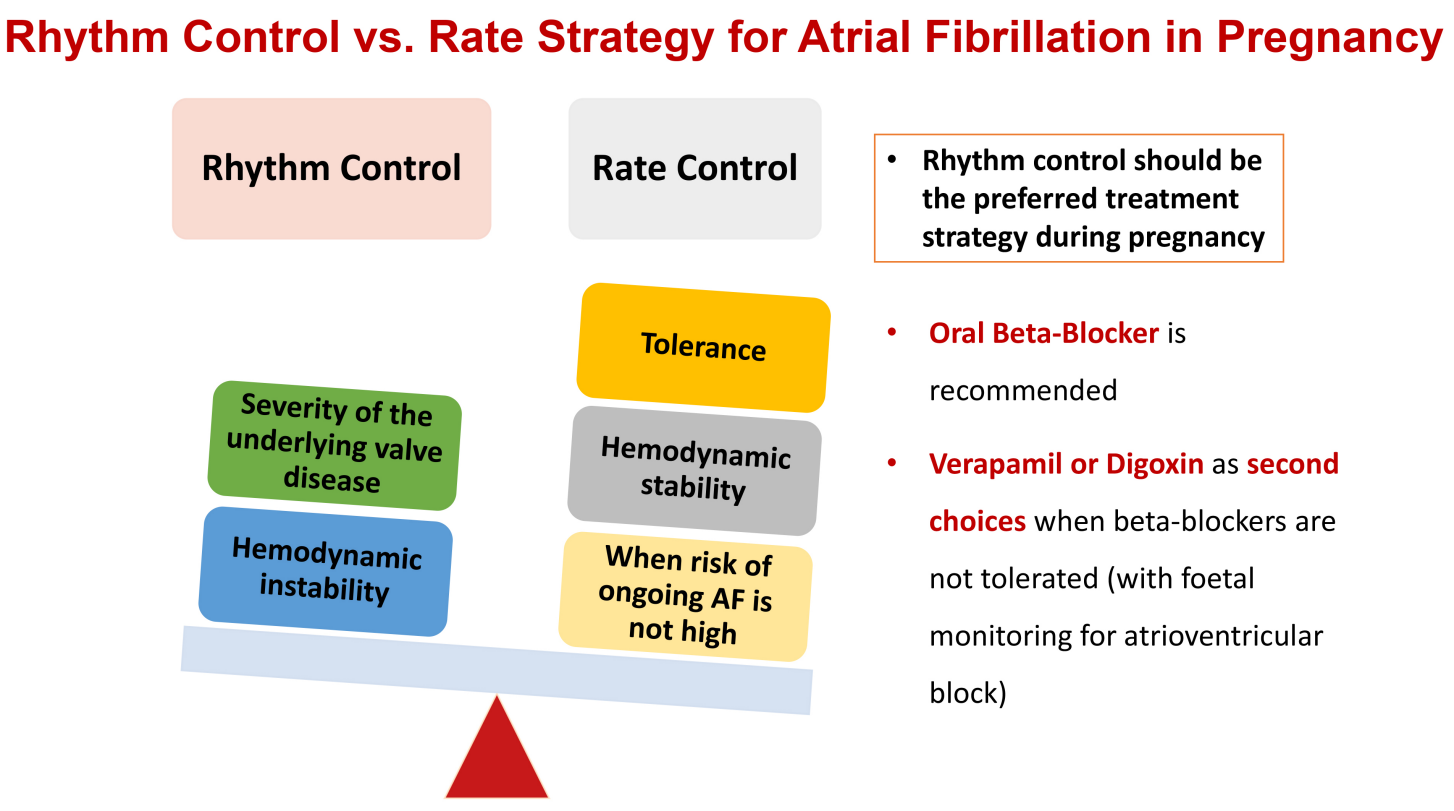
**Rhythm control and rate strategy for atrial fibrillation (AF) in pregnancy. **Rhythm control should be the preferred treatment strategy during pregnancy. If rate control is chosen, β-blockers should be the first line of therapy, with digoxin, verapamil or diltiazem as the second choice.

Irrespective of the coexistence of structural heart disease, AF in pregnancy may be benign and self-limited, or it may represent a hemodynamically significant condition. Some patients with AF spontaneously convert to SR without requiring medical therapy, although pharmacological or electrical cardioversion (ECV) may be necessary. The combination of rapid ventricular response and loss of effective atrial contraction, which typically accounts for 15–20% of left ventricular filling volume, may cause hemodynamic instability. Indeed, a shortened diastolic filling time due to rapid ventricular response reduces cardiac output. This can lead to maternal systemic hypoperfusion which adversely affects fetal circulation. A reduction in blood pressure due to tachycardia can result in fetal bradycardia and warrant urgent intervention with ECV, drugs, or emergency cesarean section. Therefore, prompt detection and early management of AF can prevent fetal and maternal complications. Rhythm control should be the preferred treatment strategy during pregnancy [4] (Fig. 3). ECV should be performed promptly in all situations in which reduced uterine blood flow and/or hemodynamic instability endangers the safety of the mother or the fetus [2].

**Fig. 4.**
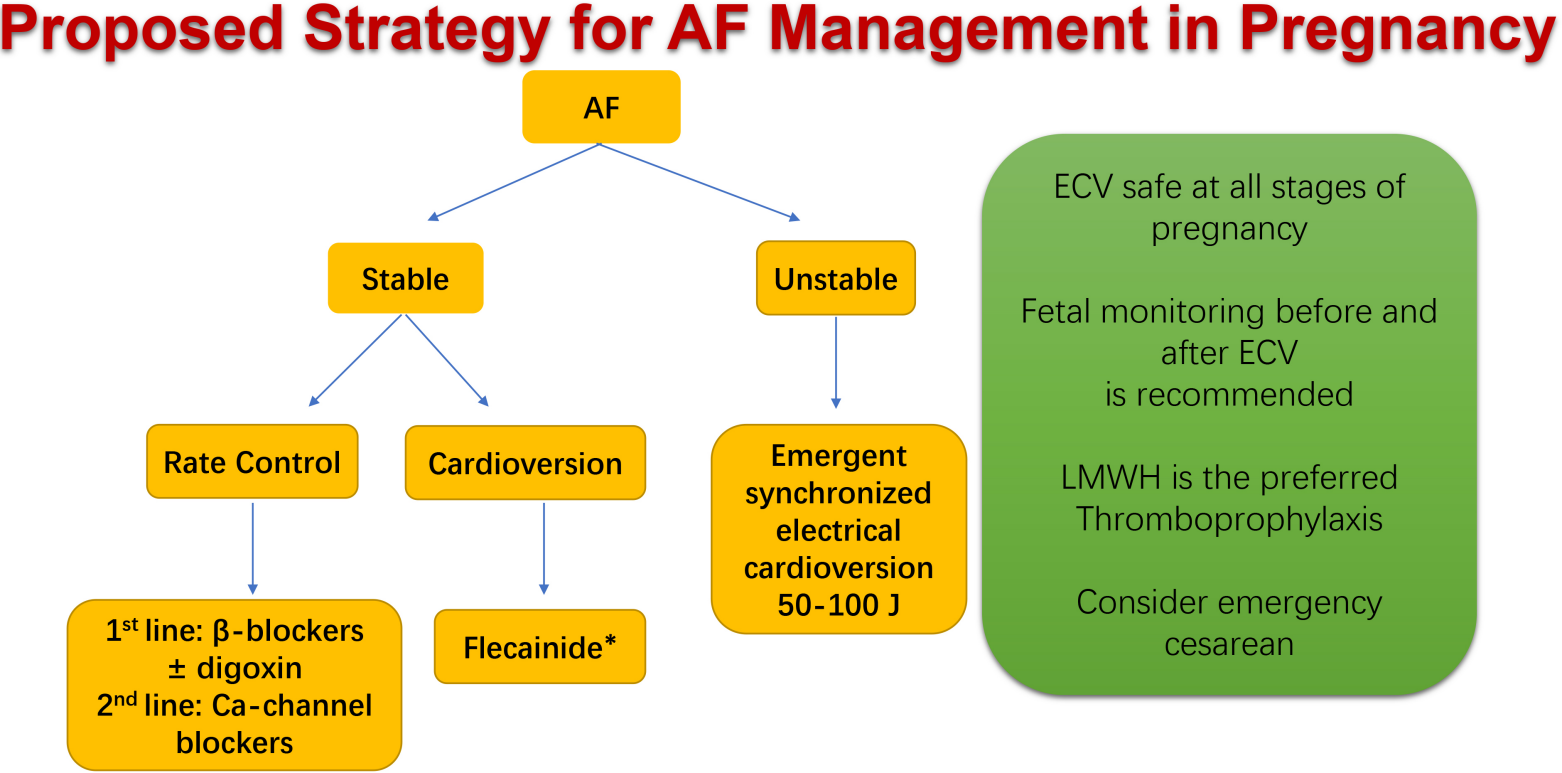
**Proposed strategy for atrial fibrillation (AF) management in pregnancy. **Hemodynamic condition is the most important factor for determining the appropriate management of AF in pregnancy. Electrical cardioversion (ECV) should be performed promptly if there is hemodynamic instability or if the arrhythmias present a risk to the mother and/or fetus. The ECV option may also be considered for stable patients. ECV during pregnancy is relatively safe at all stages of pregnancy when using a synchronized external direct current (50–100 J biphasic shocks for AF, and 25–50 J for atrial flutter), and with monitoring of the fetal heart rate during cardioversion. In stable patients with structurally normal hearts, a pharmacologic cardioversion attempt can be performed safely using intravenous flecainide [71]. *Flecainide is relatively contraindicated in women with structural heart disease, and is also contraindicated in case of atrial flutter due to risk of 1:1 AV conduction. LMWH, low-molecular-weight-heparin; AV, aortic valve.

Randomized controlled studies on the use of antiarrhythmic drugs (AADs) during pregnancy are lacking. According to the latest European Society of Cardiology (ESC) guidelines on AF management, ECV is recommended for patients who are hemodynamically unstable or have a pre-existing AF (Class I, Level C) [70] (Fig. 4, Ref. [71]). If hypertrophic cardiomyopathy (HCM) coexists, the option of ECV should be considered for persistent AF conversion (Class II, level A) [70].

ECV is considered relatively safe during all stages of pregnancy, since only a small amount of current reaches the uterus [20]. External direct current synchronized ECV using 50–100 J biphasic shocks for AF and 25–50 J for atrial flutter is usually successful [59, 70, 72]. In some case reports, the ECV was repeated more than once in pregnant women with good results [70, 72].

However, due to the lack of clinical studies, ECV should only be carried out when deemed absolutely necessary [59]. It has been suggested that ECV has low risks for the induction of uterine contractions [2, 7], fetal arrhythmias, and preterm labor [2, 73]. Fetal HR should be closely monitored during ECV so as to rapidly manage any potential adverse effects [59]. Facilities for emergency cesarean section should also be available [74]. Cardioversion should generally be preceded by anticoagulation, whilst intravenous β-blockers are recommended for initial acute rate control [70, 75]. Sedation during ECV can be performed using propofol, which is chosen due to its rapid onset, short duration, and safety in pregnancy. Propofol doses of 2 mg/kg body weight appear to have no negative impact, but high doses can cause fetal respiratory depression or even asphyxia [76]. AADs should be avoided whenever possible during pregnancy, as they can cross the placenta and may adversely affect fetal development and fetal heart rhythm [77]. However, pharmacologic acute rhythm control can be attempted in stable patients with structurally normal hearts [4, 70]. In such cases, intravenous flecainide or ibutilide can be used safely for pharmacological conversion [70, 76, 78, 79]. For cases with underlying structural heart disease and recent arrhythmic onset, ECV is considered to be the safest treatment option [4, 70].

Amiodarone can cause many adverse fetal effects including hypothyroidism and delayed growth. It is classified in the class D pregnancy risk category according to the Food and Drug Administration (FDA) [80, 81, 82]. Therefore, it should only be used for emergency situations in pregnant women.

Following cardioversion, the use of oral AADs such as flecainide, propafenone or sotalol should be considered in order to maintain SR and to prevent AF recurrence in the event that atrio-ventricular nodal (AVN) blocking drugs fail [4, 70]. Amiodarone is not recommended for long-term rhythm control in pregnancy (class III) [70]. Catheter ablation (CA) (radiofrequency or cryoablation) may be considered for the management of poorly tolerated and drug-resistant arrhythmias [83]. However, the risk of fetal radiation exposure must be taken into account with CA, especially during the early stages of pregnancy. Even when the advantages of CA are expected to outweigh the disadvantages, it is important to minimize fetal radiation exposure and thus protect organogenesis and neurodevelopment.

Electro-anatomic mapping and intracardiac echocardiography can lower the exposure to ionizing radiation. It is possible to achieve reliable three-dimensional (3D) geometrical mapping of the left atrium (LA) using non-fluoroscopic-based electroanatomical systems [84]. Atrial or atrioventricular re-entrant tachycardia can thus be treated safely during pregnancy using an electroanatomical mapping system, although the data is still limited [85, 86, 87, 88, 89, 90, 91]. Conversely, CA of AF/AFL during pregnancy is generally not recommended [4, 68, 70]. Although CA may be considered in refractory symptomatic patients, it is advisable to defer the procedure until the post-partum period. However, a zero-fluoroscopy approach may be considered for resistant cases [84]. Moreover, arrhythmia ablation may in certain cases be considered before pregnancy.

## 7. Rate Control

In view of the limited data available for verapamil and diltiazem use, ESC guidelines recommend the use of β-blockers as first-line treatment for acute and/or long-term rate control during pregnancy [4, 92]. β-blockers are also the first-line medication for hypertension during pregnancy and are generally considered to be safe [2, 4, 70, 93]. In pregnancies that are complicated by hypertension and treated with propranolol, no apparent congenital anomalies were observed, but growth retardation was reported [59, 77, 94]. The use of atenolol in the first trimester has also been associated with delayed fetal growth [59, 77, 92, 95, 96, 97]. β1-selective beta-blockers (metoprolol, bisoprolol) are the recommended first choice to prevent β2-mediated peripheral vasodilation, uterine relaxation, and fetal hypoglycemia [59, 77, 92, 98]. Digoxin or Ca-channel blockers should be considered for rate control if the beta-blockers fail [70, 92]. Digoxin has not been associated with teratogenic effects and is also useful as a rate-control agent [70, 77]. However, digoxin crosses the placenta, and fetal death has been reported in extreme cases of maternal digitalis intoxication [99]. Of note, digoxin blood levels may be unpredictable during pregnancy due to interference with immunoreactive serum components and potential drug interactions, so careful monitoring is mandatory [59, 77, 98]. The use of non-dihydropyridine calcium-channel blockers is generally considered for second-line therapy of rate control in AF [70]. The use of diltiazem in pregnancy is not recommended because animal studies have revealed evidence of teratogenicity [70, 100]. Verapamil is considered safer than diltiazem [101] and with precautions it can be used as a second-line choice [70, 92]. However, intravenous administration of verapamil may cause maternal hypotension and subsequent fetal distress, bradycardia, and high-degree AV block. This formulation should therefore be avoided during the first two trimesters of pregnancy [59, 77, 98]. Beta-blockers, class IC AAD, and sotalol should be used with caution if systemic ventricular function is impaired [59, 77, 98]. Table 1 lists the adverse effects of the AADs that are commonly used for rhythm and rate control during pregnancy. Finally, CA (radiofrequency or cryoablation) prior to pregnancy may be considered in select cases to prevent AF during pregnancy [2].

**Table 1.** **Antiarrhythmic drugs (AADs): FDA classification and adverse effects in pregnancy**.

	FDA category	Placenta permeability	Adverse effects
Amiodarone	D	Yes	Thyroid insufficiency, hyperthyroidism, goiter, bradycardia, growth retardation, premature birth.
Atenolol	D	Yes	Hypospadias (first trimester); birth defects, low birth weight, bradycardia and hypoglycaemia in fetus (second and third trimester).
Bisoprolol	C	Yes	Bradycardia and hypoglycaemia in fetus.
Digoxin	C	Yes	Bradycardia and hypoglycaemia in fetus.
Diltiazem	C	No	Possible teratogenic effects.
Flecainide	C	Yes	Unknown
Labetalol	C	Yes	Intrauterine growth retardation (second and third trimester), neonatal bradycardia and hypotension (used near term).
Propafenone	C	Yes	Unknown
Propranolol	C	Yes	Bradycardia and hypoglycaemia in fetus.
Sotalol	B	Yes	Bradycardia and hypoglycaemia in fetus.
Verapamil oral	C	Yes	Well tolerated
Verapamil IV	C	Yes	Risk of hypotension and subsequent fetal hypoperfusion.

The most frequently reported adverse effects of antiarrhythmic drugs (AADs) used for rhythm and rate control during pregnancy are shown in Table 1. The risk category for each drug according to the Food and Drug Administration (FDA) classification is also shown. Category A: No risk has been reported in human studies. The drug appears to be safe for the fetus during the first trimester. Category B: No risks have been found in experimental studies. Category C: Risk cannot be excluded. Although no risk for the fetus was found in animal studies, there are insufficient studies in pregnant women. Category D: A risk for the fetus has been reported in studies on pregnant women. Category X: The drug is contraindicated.

## 8. Anticoagulation

It is well-known that pregnancy represents a prothrombotic condition. This is due to changes in hemostasis that cause physiological hypercoagulability, thus protecting women from possible hemorrhage during delivery [14]. A 5-fold increased risk of venous thromboembolism (VTE) has been reported during pregnancy [102], and the risk of thrombosis remains high for three months after partum [103].

However, the data so far on the risk of stroke and AF in pregnant women is quite limited.

Thrombotic and embolic risk stratification in pregnant women is similar to that of non-pregnant women, since pregnancy is not included as a risk factor in the commonly used scores [70]. Moreover, the ${\mathrm{CHA}}_{2}$${\mathrm{DS}}_{2}$-VASC has not been validated for pregnant women and is thought to underestimate the risk of stroke in pregnant females with AF [104, 105, 106]. Nevertheless, it is currently the only score system recommended for pregnant women [4].

According to the latest European Guidelines [4], the same criteria used to stratify stroke risk in non-pregnant females should also be applied for pregnant women. Consequently, the onus is on physicians to consider the risk of thromboembolism in pregnant women with AF and to choose the most appropriate anticoagulation strategy that safely balances maternal and fetal risks [107]. When mitral stenosis is present, a full anticoagulation strategy is required. Moreover, patients with hypertrophic cardiomyopathy (HCM) and AF are more likely to develop thromboembolic events [108]. According to the American College of Cardiology/American Heart Association (ACC/AHA) guidelines for HCM, it is advisable to anticoagulate pregnant females, regardless of their ${\mathrm{CHA}}_{2}$${\mathrm{DS}}_{2}$-VASc score [109]. However, results on anticoagulation for AF during pregnancy are still lacking, and have been deduced mainly from pregnant patients with mechanical prosthetic valves [2].

Unfractionated heparin (UFH) or low-molecular-weight-heparin (LMWH) are the preferred anticoagulants in pregnant women [4] due to their inability to cross the placenta [110]. However, they have several disadvantages including the need for multiple injections and frequent monitoring [110, 111].

Vitamin K antagonists (VKA) can cross the placenta [112], leading to a 0.6%–10% incidence of embryopathies such as limb defects and nasal hypoplasia [113], and a 0.7%–2% incidence of fetopathies such as ocular defects, central nervous system abnormalities, and intracranial hemorrhage [114] during the first and second-third trimesters, respectively. VKA teratogenicity is dose-dependent, with an incidence of 0.45%–0.9% for low-dose warfarin [115]. Therefore, if low-dose VKA (warfarin <5 mg/day, phenprocoumon <3 mg/day, or acenocoumarol <2 mg/day) [116] is sufficient to achieve the target therapeutic international normalised ratio (INR) for AF, treatment may be continued during the first trimester with a low risk of toxicity [117, 118].

If the target therapeutic INR is not achieved, the VKA should be interrupted at 6–12 weeks and replaced with UFH or LMWH [68]. INR should be monitored weekly or every 2 weeks during treatment with VKA. In pregnant women treated with UFH/LMWH, the anti-Xa level and activated plasma thromboplastin time (aPTT) should be monitored weekly and aPTT prolongation of more than twice the control should be maintained [4]. According to the latest ESC guidelines, a daily warfarin intake of >5 mg/day is allowed during the second trimester (class IIa recommendation) [4]. VKAs should be stopped at the 36th week and replaced with adjusted-dose UFH/LMWH until delivery [68]. ESC guidelines also recommend replacing LMWH with intravenous UFH at least 36 h before planned vaginal delivery in moderate- and high-risk women (e.g., women with AF and prosthetic heart valves) in order to maintain aPTT value more than twice the control [4]. In the absence of bleeding complications, UFH infusion should be interrupted 4–6 h before delivery and restarted 4–6 h after delivery [4]. Therapeutic LMWH can be omitted for 24 h prior to delivery in women at low risk. For women with a planned cesarean section, LMWH can be interrupted 24 h prior to surgery, with UFH restarted at 6 h post-delivery for women at high-risk, and LMWH for women at moderate- or low-risk [4].

There is currently very little data on fetal exposure to direct-acting oral anticoagulants (DOACs) [119, 120, 121]. DOACs have been shown to pass through the placenta, although the risk of fetal bleeding has not yet been determined [119, 122, 123]. Because rivaroxaban, dabigatran, apixaban, and edoxaban have potentially toxic effects during pregnancy [124, 125, 126], DOACs are not indicated during pregnancy [4, 51].

## 9. Clinical Perspectives and Challenges Regarding AF during Pregnancy

Prompt recognition of AF during pregnancy is crucial for reducing mortality and morbidity for both mother and fetus [68]. However, the management of AF during pregnancy is complex.

Firstly, an accurate workout is required to determine the presence of structural heart disease, pulmonary embolism, pre-excitation syndrome, and alcohol or drug consumption. Circulating electrolyte levels and thyroid function should also be evaluated [68]. Moreover, the approach to management changes if there are any underlying disorders due to the different outcomes [68]. If there is coexisting valvulopathy, the development of AF may increase the risk of acute HF, especially in the first three months. The risk of hemodynamic impairment must also be carefully evaluated to avoid adverse consequences for the mother and fetus. Moreover, anti-arrhythmic and anti-coagulation therapies should be used cautiously. Follow-up during pregnancy should be performed by a Pregnancy Heart Team (PHT), or Cardio-Obstetric Team, composed of experienced cardiologists, gynecologists, anesthesiologists, obstetricians and nurses, with at least one visit per trimester [127, 128, 129]. The main aim is to achieve both maternal and fetal safety. Timely interventions may be necessary to ensure optimal fetal well-being, even in the absence of underlying heart disease. The approach must be guided by the gestational age, and the potential teratogenic effects of medications should be carefully considered. The aim of the PHT should be to provide women with comprehensive counselling, careful planning of the delivery time and modality, and close postpartum follow-up.

## 10. Summary

∙ Atrial fibrillation (AF) during pregnancy has an incidence of 27/100,000

∙ The AF incidence can be up to 39.2% in the presence of structural heart disease

∙ AF is often benign and self-limiting in women with normal hearts

∙ Both maternal and fetal risks must be assessed

∙ Electrical cardioversion (ECV) is recommended in patients who are hemodynamically unstable

∙ ECV is recommended in patients with a pre-excited AF

∙ ECV may be considered in patients with hypertrophic cardiomyopathy

∙ Antiarrhythmic drugs should be avoided during pregnancy

∙ Ablation using a zero-fluoroscopy approach is feasible for the most resistant cases

∙β-blockers should be the first-line treatment for rate control

∙ Although the ${\mathrm{CHA}}_{2}$${\mathrm{DS}}_{2}$-VASc score has been not validated in pregnant women, it is the only score that is recommended and is often very low

∙ Unfractionated heparin (UFH) or low-molecular-weight-heparin (LMWH) are the preferred anticoagulants

∙ OAC is critical, and therefore it is often better to restore sinus rhythm in order to avoid long-term OAC

## 11. Conclusions

A wide range in incidence is often reported for AF during pregnancy. This can be up to 39% if structural cardiac disorders are also present [2]. Possible underlying causes for AF should always be investigated, including thyroid disorders, electrolyte imbalance, pulmonary embolism, alcohol abuse, CHDs and cardiomyopathies. The management of AF in pregnant women can be particularly challenging. Both maternal and fetal risks must be borne in mind when choosing the most appropriate therapeutic strategy. Drug choices should be considered carefully, as well as the performance of ECV. A PHT consisting of several professional members has been proposed to improve the management of pregnant women in complex clinical contexts. A multidisciplinary team-based approach is likely to be useful for decision-making in pregnant women with AF. Further studies in this field should lead to better management of pregnant women with AF.

## References

[1] Brundel BJJM, Ai X, Hills MT, Kuipers MF, Lip GYH, de Groot NMS (2022). Atrial fibrillation. *Nature Reviews: Disease Primers*.

[2] Tamirisa KP, Elkayam U, Briller JE, Mason PK, Pillarisetti J, Merchant FM (2022). Arrhythmias in Pregnancy. *JACC: Clinical Electrophysiology*.

[3] Vaidya VR, Arora S, Patel N, Badheka AO, Patel N, Agnihotri K (2017). Burden of Arrhythmia in Pregnancy. *Circulation*.

[4] Regitz-Zagrosek V, Roos-Hesselink JW, Bauersachs J, Blomström-Lundqvist C, Cífková R, De Bonis M (2018). 2018 ESC Guidelines for the management of cardiovascular diseases during pregnancy. *European Heart Journal*.

[5] Greenwood JP, Scott EM, Stoker JB, Walker JJ, Mary DA (2001). Sympathetic neural mechanisms in normal and hypertensive pregnancy in humans. *Circulation*.

[6] Wong AYH, Kulandavelu S, Whiteley KJ, Qu D, Langille BL, Adamson SL (2002). Maternal cardiovascular changes during pregnancy and postpartum in mice. *American Journal of Physiology-Heart and Circulatory Physiology*.

[7] DiCarlo-Meacham LA, Dahlke LJ (2011). Atrial fibrillation in pregnancy. *Obstetrics and Gynecology*.

[8] Fürniss HE, Stiller B (2021). Arrhythmic risk during pregnancy in patients with congenital heart disease. *Herzschrittmachertherapie & Elektrophysiologie*.

[9] Williams DS, Mikhova K, Sodhi S (2021). Arrhythmias and Pregnancy: Management of Preexisting and New-Onset Maternal Arrhythmias. *Cardiology Clinics*.

[10] Manolis TA, Manolis AA, Apostolopoulos EJ, Papatheou D, Melita H, Manolis AS (2020). Cardiac arrhythmias in pregnant women: need for mother and offspring protection. *Current Medical Research and Opinion*.

[11] Creanga AA, Syverson C, Seed K, Callaghan WM (2017). Pregnancy-Related Mortality in the United States, 2011–2013. *Obstetrics and Gynecology*.

[12] Lima FV, Yang J, Xu J, Stergiopoulos K (2017). National Trends and In-Hospital Outcomes in Pregnant Women With Heart Disease in the United States. *The American Journal of Cardiology*.

[13] Writing Committee Members, ACC/AHA Joint Committee Members (2022). 2022 AHA/ACC/HFSA Guideline for the Management of Heart Failure. *Journal of Cardiac Failure*.

[14] Goland S, Elkayam U (2012). Anticoagulation in pregnancy. *Cardiology Clinics*.

[15] Elkayam U, Goland S, Pieper PG, Silverside CK (2016). High-Risk Cardiac Disease in Pregnancy: Part I. *Journal of the American College of Cardiology*.

[16] Salam AM, Ertekin E, van Hagen IM, Al Suwaidi J, Ruys TPE, Johnson MR (2015). Atrial Fibrillation or Flutter During Pregnancy in Patients With Structural Heart Disease: Data From the ROPAC (Registry on Pregnancy and Cardiac Disease). *JACC. Clinical Electrophysiology*.

[17] MacIntyre C, Iwuala C, Parkash R (2018). Cardiac Arrhythmias and Pregnancy. *Current Treatment Options in Cardiovascular Medicine*.

[18] Ramlakhan KP, Kauling RM, Schenkelaars N, Segers D, Yap SC, Post MC (2022). Supraventricular arrhythmia in pregnancy. *Heart*.

[19] Lee MS, Chen W, Zhang Z, Duan L, Ng A, Spencer HT (2016). Atrial Fibrillation and Atrial Flutter in Pregnant Women-A Population-Based Study. *Journal of the American Heart Association*.

[20] Sengheiser CJ, Channer KC (2011). Recurrent atrial flutter and fibrillation in pregnancy. *BMJ Case Reports*.

[21] Scantlebury DC, Kattah AG, Weissgerber TL, Agarwal S, Mielke MM, Weaver AL (2018). Impact of a History of Hypertension in Pregnancy on Later Diagnosis of Atrial Fibrillation. *Journal of the American Heart Association*.

[22] Lucà F, Abrignani MG, Parrini I, Di Fusco SA, Giubilato S, Rao CM (2022). Update on Management of Cardiovascular Diseases in Women. *Journal of Clinical Medicine*.

[23] Oliver R, Nama VV, Howard RJ, Nikookam KH (2007). Leucopenia and atrial fibrillation: rare presentations of thyrotoxicosis in the first trimester. *Journal of Obstetrics and Gynaecology*.

[24] Chauveau S, Le Vavasseur O, Morel E, Dulac A, Chevalier P (2019). Flecainide is a safe and effective treatment for pre-excited atrial fibrillation rapidly conducted to the ventricle in pregnant women: a case series. *European Heart Journal-Case Reports*.

[25] Sauvé N, Rey É, Cumyn A, Groupe d’ÉtudeenMédecineObstétricale du Québec (2017). Atrial Fibrillation in a Structurally Normal Heart during Pregnancy: A Review of Cases From a Registry and From the Literature. *Journal of Obstetrics and Gynaecology Canada*.

[26] Gowda RM, Khan IA, Mehta NJ, Vasavada BC, Sacchi TJ (2003). Cardiac arrhythmias in pregnancy: clinical and therapeutic considerations. *International Journal of Cardiology*.

[27] Kuczkowski KM (2004). New onset transient lone atrial fibrillation in a healthy parturient: déjà vu. *International Journal of Cardiology*.

[28] Walsh CA, Manias T, Patient C (2008). Atrial fibrillation in pregnancy. *European Journal of Obstetrics, Gynecology, and Reproductive Biology*.

[29] Anugu VR, Nalluri N, Asti D, Gaddam S, Vazzana T, Lafferty J (2016). New-onset lone atrial fibrillation in pregnancy. *Therapeutic Advances in Cardiovascular Disease*.

[30] Janjua NB, Birmani SA, McDonagh T, Hameed A, McKernan M (2020). New-onset lone maternal atrial fibrillation: A case report. *Medicine*.

[31] Gelsomino S, La Meir M, Lucà F, Lorusso R, Crudeli E, Vasquez L (2012). Treatment of lone atrial fibrillation: a look at the past, a view of the present and a glance at the future. *European Journal of Cardio-Thoracic Surgery*.

[32] Cumyn A, Sauvé N, Rey É (2017). Atrial fibrillation with a structurally normal heart in pregnancy: An international survey on current practice. *Obstetric Medicine*.

[33] Huisman CM, Zwart JJ, Roos-Hesselink JW, Duvekot JJ, van Roosmalen J (2013). Incidence and predictors of maternal cardiovascular mortality and severe morbidity in The Netherlands: a prospective cohort study. *PLoS One*.

[34] Chokesuwattanaskul R, Thongprayoon C, Bathini T, O’Corragain OA, Sharma K, Prechawat S (2019). Incidence of atrial fibrillation in pregnancy and clinical significance: A meta-analysis. *Advances in Medical Sciences*.

[35] Leśniak-Sobelga A, Tracz W, KostKiewicz M, Podolec P, Pasowicz M (2004). Clinical and echocardiographic assessment of pregnant women with valvular heart diseases–maternal and fetal outcome. *International Journal of Cardiology*.

[36] Autore C, Conte MR, Piccininno M, Bernabò P, Bonfiglio G, Bruzzi P (2002). Risk associated with pregnancy in hypertrophic cardiomyopathy. *Journal of the American College of Cardiology*.

[37] Silversides CK, Harris L, Haberer K, Sermer M, Colman JM, Siu SC (2006). Recurrence rates of arrhythmias during pregnancy in women with previous tachyarrhythmia and impact on fetal and neonatal outcomes. *The American Journal of Cardiology*.

[38] Lin CH, Lee CN (2008). Atrial fibrillation with rapid ventricular response in pregnancy. *Taiwanese Journal of Obstetrics & Gynecology*.

[39] Walker MG, Colman J, Silversides CK, Gandhi S, Kingdom J (2011). Maternal atrial arrhythmia detected by uterine artery Doppler. *Journal of Obstetrics and Gynaecology Canada*.

[40] Malhotra M, Sharma JB, Tripathii R, Arora P, Arora R (2004). Maternal and fetal outcome in valvular heart disease. *International Journal of Gynaecology and Obstetrics*.

[41] Ferrero S, Colombo BM, Ragni N (2004). Maternal arrhythmias during pregnancy. *Archives of Gynecology and Obstetrics*.

[42] Diker E, Aydogdu S, Ozdemir M, Kural T, Polat K, Cehreli S (1996). Prevalence and predictors of atrial fibrillation in rheumatic valvular heart disease. *The American Journal of Cardiology*.

[43] Shankar P R B, Roa B H, Jaishankar S, Narasimhan M (2010). Current Perspectives: Rheumatic Atrial Fibrillation. *Journal of Atrial Fibrillation*.

[44] Sharma SK, Verma SH (2015). A Clinical Evaluation of Atrial Fibrillation in Rheumatic Heart Disease. *The Journal of the Association of Physicians of India*.

[45] Szekely P, Snaith L (1961). Atrial fibrillation and pregnancy. *British Medical Journal*.

[46] Khairy P, Ouyang DW, Fernandes SM, Lee-Parritz A, Economy KE, Landzberg MJ (2006). Pregnancy outcomes in women with congenital heart disease. *Circulation*.

[47] Nqayana T, Moodley J, Naidoo DP (2008). Cardiac disease in pregnancy. *Cardiovascular Journal of Africa*.

[48] Parasuraman R, Gandhi MM, Liversedge NH (2006). Nifedipine tocolysis associated atrial fibrillation responds to DC cardioversion. *BJOG*.

[49] Alsagaff MY, Oktaviono YH, Dharmadjati BB, Lefi A, Al-Farabi MJ, Gandi P (2021). Electrocardiography on admission is associated with poor outcomes in coronavirus disease 2019 (COVID-19) patients: A systematic review and meta-analysis. *Journal of Arrhythmia*.

[50] Nishiga M, Wang DW, Han Y, Lewis DB, Wu JC (2020). COVID-19 and cardiovascular disease: from basic mechanisms to clinical perspectives. *Nature Reviews. Cardiology*.

[51] Mehta LS, Warnes CA, Bradley E, Burton T, Economy K, Mehran R (2020). Cardiovascular Considerations in Caring for Pregnant Patients: A Scientific Statement From the American Heart Association. *Circulation*.

[52] Liu LX, Arany Z (2014). Maternal cardiac metabolism in pregnancy. *Cardiovascular Research*.

[53] Sanghavi M, Rutherford JD (2014). Cardiovascular physiology of pregnancy. *Circulation*.

[54] Nakagaki A, Inami T, Minoura T, Baba R, Iwase S, Sato M (2016). Differences in autonomic neural activity during exercise between the second and third trimesters of pregnancy. *The Journal of Obstetrics and Gynaecology Research*.

[55] Shotan A, Ostrzega E, Mehra A, Johnson JV, Elkayam U (1997). Incidence of arrhythmias in normal pregnancy and relation to palpitations, dizziness, and syncope. *The American Journal of Cardiology*.

[56] Chapman AB, Abraham WT, Zamudio S, Coffin C, Merouani A, Young D (1998). Temporal relationships between hormonal and hemodynamic changes in early human pregnancy. *Kidney International*.

[57] Chesley LC (1972). Plasma and red cell volumes during pregnancy. *American Journal of Obstetrics and Gynecology*.

[58] Gowda RM, Khan IA, Mehta NJ, Vasavada BC, Sacchi TJ (2003). Cardiac arrhythmias in pregnancy: clinical and therapeutic considerations. *International Journal of Cardiology*.

[59] Enriquez AD, Economy KE, Tedrow UB (2014). Contemporary management of arrhythmias during pregnancy. *Circulation. Arrhythmia and Electrophysiology*.

[60] Franz MR, Cima R, Wang D, Profitt D, Kurz R (1992). Electrophysiological effects of myocardial stretch and mechanical determinants of stretch-activated arrhythmias. *Circulation*.

[61] Yan Y, Skarsfeldt MA, Diness JG, Bentzen BH (2021). Small conductance calcium activated K^+^ channel inhibitor decreases stretch induced vulnerability to atrial fibrillation. *International Journal of Cardiology. Heart & Vasculature*.

[62] Roberts JM, Insel PA, Goldfien A (1981). Regulation of myometrial adrenoreceptors and adrenergic response by sex steroids. *Molecular Pharmacology*.

[63] Kodogo V, Azibani F, Sliwa K (2019). Role of pregnancy hormones and hormonal interaction on the maternal cardiovascular system: a literature review. *Clinical Research in Cardiology*.

[64] Long V, Fiset C (2020). Contribution of estrogen to the pregnancy-induced increase in cardiac automaticity. *Journal of Molecular and Cellular Cardiology*.

[65] El Khoury N, Ross JL, Long V, Thibault S, Ethier N, Fiset C (2018). Pregnancy and oestrogen regulate sinoatrial node calcium homeostasis and accelerate pacemaking. *Cardiovascular Research*.

[66] Conrad KP (2011). Emerging role of relaxin in the maternal adaptations to normal pregnancy: implications for preeclampsia. *Seminars in Nephrology*.

[67] Leffert LR, Clancy CR, Bateman BT, Bryant AS, Kuklina EV (2015). Hypertensive disorders and pregnancy-related stroke: frequency, trends, risk factors, and outcomes. *Obstetrics and Gynecology*.

[68] Youssef GS (2019). Management of atrial fibrillation during pregnancy. *E-Journal of Cardiology Practice*.

[69] Lee JS, Choi ES, Hwang Y, Lee KS, Ahn KH (2023). Preterm birth and maternal heart disease: A machine learning analysis using the Korean national health insurance database. *PLoS One*.

[70] Hindricks G, Potpara T, Dagres N, Arbelo E, Bax JJ, Blomström-Lundqvist C (2021). 2020 ESC Guidelines for the diagnosis and management of atrial fibrillation developed in collaboration with the European Association for Cardio-Thoracic Surgery (EACTS): The Task Force for the diagnosis and management of atrial fibrillation of the European Society of Cardiology (ESC) Developed with the special contribution of the European Heart Rhythm Association (EHRA) of the ESC. *European Heart Journal*.

[71] Lucà F, Giubilato S, Di Fusco SA, Piccioni L, Rao CM, Iorio A (2021). Anticoagulation in atrial fibrillation cardioversion: what is crucial to take into account. *Journal of Clinical Medicine*.

[72] Tromp CHN, Nanne ACM, Pernet PJM, Tukkie R, Bolte AC (2011). Electrical cardioversion during pregnancy: safe or not. *Netherlands Heart Journal*.

[73] Page RL (1995). Treatment of arrhythmias during pregnancy. *American Heart Journal*.

[74] Barnes EJ, Eben F, Patterson D (2002). Direct current cardioversion during pregnancy should be performed with facilities available for fetal monitoring and emergency caesarean section. *BJOG*.

[75] Kirchhof P, Benussi S, Kotecha D, Ahlsson A, Atar D, Casadei B (2016). 2016 ESC Guidelines for the management of atrial fibrillation developed in collaboration with EACTS. *European Journal of Cardio-thoracic Surgery*.

[76] Iliodromitis K, Kociszewski J, Bogossian H (2021). Atrial fibrillation during pregnancy: a 9-month period with limited options. *Herzschrittmachertherapie & Elektrophysiologie*.

[77] Halpern DG, Weinberg CR, Pinnelas R, Mehta-Lee S, Economy KE, Valente AM (2019). Use of Medication for Cardiovascular Disease During Pregnancy: JACC State-of-the-Art Review. *Journal of the American College of Cardiology*.

[78] Katritsis DG, Boriani G, Cosio FG, Hindricks G, Jaïs P, Josephson ME (2017). European Heart Rhythm Association (EHRA) consensus document on the management of supraventricular arrhythmias, endorsed by Heart Rhythm Society (HRS), Asia-Pacific Heart Rhythm Society (APHRS), and Sociedad Latinoamericana de EstimulaciónCardiaca y Electrofisiologia (SOLAECE). *Europace*.

[79] Elkayam U, Jalnapurkar S, Barakkat MN, Khatri N, Kealey AJ, Mehra A (2014). Pregnancy-associated acute myocardial infarction: a review of contemporary experience in 150 cases between 2006 and 2011. *Circulation*.

[80] Bartalena L, Bogazzi F, Braverman LE, Martino E (2001). Effects of amiodarone administration during pregnancy on neonatal thyroid function and subsequent neurodevelopment. *Journal of Endocrinological Investigation*.

[81] Park K, Bairey Merz CN, Bello NA, Davis M, Duvernoy C, Elgendy IY (2021). Management of Women With Acquired Cardiovascular Disease From Pre-Conception Through Pregnancy and Postpartum: JACC Focus Seminar 3/5. *Journal of the American College of Cardiology*.

[82] Addis A, Sharabi S, Bonati M (2000). Risk classification systems for drug use during pregnancy: are they a reliable source of information. *Drug Safety*.

[83] Best PJM, Skelding KA, Mehran R, Chieffo A, Kunadian V, Madan M (2011). SCAI consensus document on occupational radiation exposure to the pregnant cardiologist and technical personnel. *Catheterization and Cardiovascular Interventions*.

[84] Di Cori A, Zucchelli G, Faggioni L, Segreti L, De Lucia R, Barletta V (2021). Role of pre-procedural CT imaging on catheter ablation in patients with atrial fibrillation: procedural outcomes and radiological exposure. *Journal of Interventional Cardiac Electrophysiology*.

[85] Risius T, Mortensen K, Meinertz T, Willems S (2008). Cluster of multiple atrial tachycardias limited to pregnancy after radiofrequency ablation following senning operation. *International Journal of Cardiology*.

[86] Ferguson JD, Helms A, Mangrum JM, DiMarco JP (2011). Ablation of incessant left atrial tachycardia without fluoroscopy in a pregnant woman. *Journal of Cardiovascular Electrophysiology*.

[87] Szumowski L, Szufladowicz E, Orczykowski M, Bodalski R, Derejko P, Przybylski A (2010). Ablation of severe drug-resistant tachyarrhythmia during pregnancy. *Journal of Cardiovascular Electrophysiology*.

[88] Bongiorni MG, Di Cori A, Soldati E, Zucchelli G, Segreti L, Solarino G (2008). Radiofrequency catheter ablation of atrioventricular nodal reciprocating tachycardia using intracardiac echocardiography in pregnancy. *Europace*.

[89] Berruezo A, Díez GR, Berne P, Esteban M, Mont L, Brugada J (2007). Low exposure radiation with conventional guided radiofrequency catheter ablation in pregnant women. *Pacing and Clinical Electrophysiology*.

[90] Kanjwal Y, Kosinski D, Kanj M, Thomas W, Grubb B (2005). Successful radiofrequency catheter ablation of left lateral accessory pathway using transseptal approach during pregnancy. *Journal of Interventional Cardiac Electrophysiology*.

[91] Damilakis J, Theocharopoulos N, Perisinakis K, Manios E, Dimitriou P, Vardas P (2001). Conceptus radiation dose and risk from cardiac catheter ablation procedures. *Circulation*.

[92] Brugada J, Katritsis DG, Arbelo E, Arribas F, Bax JJ, Blomström-Lundqvist C (2020). 2019 ESC Guidelines for the management of patients with supraventricular tachycardiaThe Task Force for the management of patients with supraventricular tachycardia of the European Society of Cardiology (ESC). *European Heart Journal*.

[93] Kirchhof P, Benussi S, Kotecha D, Ahlsson A, Atar D, Casadei B (2016). 2016 ESC Guidelines for the management of atrial fibrillation developed in collaboration with EACTS. *European Heart Journal*.

[94] Pruyn SC, Phelan JP, Buchanan GC (1979). Long-term propranolol therapy in pregnancy: maternal and fetal outcome. *American Journal of Obstetrics and Gynecology*.

[95] Lip GY, Beevers M, Churchill D, Shaffer LM, Beevers DG (1997). Effect of atenolol on birth weight. *The American Journal of Cardiology*.

[96] Lydakis C, Lip GY, Beevers M, Beevers DG (1999). Atenolol and fetal growth in pregnancies complicated by hypertension. *American Journal of Hypertension*.

[97] Bateman BT, Patorno E, Desai RJ, Seely EW, Mogun H, Maeda A (2016). Late Pregnancy β Blocker Exposure and Risks of Neonatal Hypoglycemia and Bradycardia. *Pediatrics*.

[98] Tita AT, Szychowski JM, Boggess K, Dugoff L, Sibai B, Lawrence K (2022). Treatment for Mild Chronic Hypertension during Pregnancy. *The New England Journal of Medicine*.

[99] Rogers MC, Willerson JT, Goldblatt A, Smith TW (1972). Serum digoxin concentrations in the human fetus, neonate and infant. *The New England Journal of Medicine*.

[100] Akagi Y, Iketaki A, Nakamura R, Yamamura S, Endo M, Morikawa K (2021). Association between Cerebral Infarction Risk and Medication Adherence in Atrial Fibrillation Patients Taking Direct Oral Anticoagulants. *Healthcare*.

[101] Pieper PG (2015). Use of medication for cardiovascular disease during pregnancy. *Nature Reviews Cardiology*.

[102] James AH (2009). Venous thromboembolism in pregnancy. *Arteriosclerosis, Thrombosis, and Vascular Biology*.

[103] Kamel H, Navi BB, Sriram N, Hovsepian DA, Devereux RB, Elkind MSV (2014). Risk of a thrombotic event after the 6-week postpartum period. *The New England Journal of Medicine*.

[104] Lindley K, LoSapio D, Conner S, Billadello J, Barger P, Cahill A (2018). Evaluation of CHA2DS2-VASC risk stratification tool in pregnant women with atrial fibrillation or atrial flutter. *Journal of the American College of Cardiology*.

[105] Arasar K, Briller JE, Naksuk N (2020). Where CHA2DS2-VASC fails: thromboembolic risk with atrial fibrillation in pregnancy. *Journal of the American College of Cardiology*.

[106] Sun Y, Ling Y, Chen Z, Wang Z, Li T, Tong Q (2023). Finding low CHA2DS2-VASc scores unreliable? Why not give morphological and hemodynamic methods a try. *Frontiers in Cardiovascular Medicine*.

[107] Nichols KM, Henkin S, Creager MA (2020). Venous Thromboembolism Associated With Pregnancy: JACC Focus Seminar. *Journal of the American College of Cardiology*.

[108] Lloji A, Panza JA (2022). The Challenge of Pregnancy in Women With Hypertrophic Cardiomyopathy. *Cardiology in Review*.

[109] Ommen SR, Mital S, Burke MA, Day SM, Deswal A, Elliott P (2020). 2020 AHA/ACC Guideline for the Diagnosis and Treatment of Patients With Hypertrophic Cardiomyopathy: A Report of the American College of Cardiology/American Heart Association Joint Committee on Clinical Practice Guidelines. *Circulation*.

[110] Cacciotti L, Passaseo I (2010). Management of Atrial Fibrillation in Pregnancy. *Journal of Atrial Fibrillation*.

[111] Garcia DA, Baglin TP, Weitz JI, Samama MM (2012). Parenteral anticoagulants: Antithrombotic Therapy and Prevention of Thrombosis, 9th ed: American College of Chest Physicians Evidence-Based Clinical Practice Guidelines. *Chest*.

[112] Alshawabkeh L, Economy KE, Valente AM (2016). Anticoagulation During Pregnancy: Evolving Strategies With a Focus on Mechanical Valves. *Journal of the American College of Cardiology*.

[113] Xu Z, Fan J, Luo X, Zhang WB, Ma J, Lin YB (2016). Anticoagulation Regimens During Pregnancy in Patients With Mechanical Heart Valves: A Systematic Review and Meta-analysis. *The Canadian Journal of Cardiology*.

[114] Rutz T, Eggel-Hort B, Alberio L, Bouchardy J (2021). Anticoagulation of women with congenital heart disease during pregnancy. *International Journal of Cardiology Congenital Heart Disease*.

[115] Hassouna A, Allam H (2014). Limited dose warfarin throughout pregnancy in patients with mechanical heart valve prosthesis: a meta-analysis. *Interactive Cardiovascular and Thoracic Surgery*.

[116] Fraccaro C, Tence N, Masiero G, Karam N (2020). Management of Valvular Disease During Pregnancy: Evolving Role of Percutaneous Treatment. *Interventional Cardiology*.

[117] Mcilvaine S, Feinberg L, Spiel M (2021). Cardiovascular disease in pregnancy. *NeoReviews*.

[118] Bates SM, Rajasekhar A, Middeldorp S, McLintock C, Rodger MA, James AH (2018). American Society of Hematology 2018 guidelines for management of venous thromboembolism: venous thromboembolism in the context of pregnancy. *Blood Advances*.

[119] Beyer-Westendorf J, Michalski F, Tittl L, Middeldorp S, Cohen H, Abdul Kadir R (2016). Pregnancy outcome in patients exposed to direct oral anticoagulants - and the challenge of event reporting. *Thrombosis and Haemostasis*.

[120] Godin R, Tanguay MC (2017). The Anticoagulation Conundrum of Mechanical Heart Valves in Pregnancy: Should DOACs Be Considered. *Journal of the American College of Cardiology*.

[121] Steinberg ZL, Krieger EV (2017). Reply: The Anticoagulation Conundrum of Mechanical Heart Valves in Pregnancy: Should DOACs Be Considered. *Journal of the American College of Cardiology*.

[122] Bapat P, Pinto LSR, Lubetsky A, Aleksa K, Berger H, Koren G (2016). Examining the transplacental passage of apixaban using the dually perfused human placenta. *Journal of Thrombosis and Haemostasis*.

[123] Tomaselli GF, Mahaffey KW, Cuker A, Dobesh PP, Doherty JU, Eikelboom JW (2020). 2020 ACC Expert Consensus Decision Pathway on Management of Bleeding in Patients on Oral Anticoagulants: A Report of the American College of Cardiology Solution Set Oversight Committee. *Journal of the American College of Cardiology*.

[124] Roberti R, Iannone LF, Palleria C, Curcio A, Rossi M, Sciacqua A (2021). Direct Oral Anticoagulants: From Randomized Clinical Trials to Real-World Clinical Practice. *Frontiers in Pharmacology*.

[125] Lameijer H, Aalberts JJJ, van Veldhuisen DJ, Meijer K, Pieper PG (2018). Efficacy and safety of direct oral anticoagulants during pregnancy; a systematic literature review. *Thrombosis Research*.

[126] Lucà F, Oliva F, Abrignani MG, Di Fusco SA, Parrini I, Canale ML (2023). Management of Patients Treated with Direct Oral Anticoagulants in Clinical Practice and Challenging Scenarios. *Journal of Clinical Medicine*.

[127] Lucà F, La Meir M, Rao CM, Parise O, Vasquez L, Carella R (2011). Pharmacological management of atrial fibrillation: one, none, one hundred thousand. *Cardiology Research and Practice*.

[128] Lucà F, Colivicchi F, Parrini I, Russo MG, Di Fusco SA, Ceravolo R (2023). The role of the pregnancy heart team in clinical practice. *Frontiers in Cardiovascular Medicine*.

[129] Parrini I, Lucà F, Favilli S, Domenicucci S, Russo MG, Sarubbi B (2022). Pregnancy and heart disease: the role of the Pregnancy Heart Team. *Giornale Italiano Di Cardiologia (2006)*.

